# Longitudinal associations between alcohol use, occupational stressors, and mental health among healthcare and ancillary workers in the United Kingdom during the COVID-19 pandemic (UK-REACH)

**DOI:** 10.1186/s12916-025-04474-4

**Published:** 2025-11-28

**Authors:** Patricia Irizar, Christopher A. Martin, Katherine Woolf, Laura B. Nellums, Irtiza Qureshi, Asad Masood, Luke Bryant, Laura Goodwin, Manish Pareek, The UK-REACH Study Collaborative Group, Katherine Woolf, Laura B. Nellums, Manish Pareek, Laura Gray, Anna L Guyatt, Catherine John, I Chris McManus, Ibrahim Abubakar, Amit Gupta, Avinash Aujayeb, Bindu Gregary, Rubina Reza, Sandra Simpson, Stephen Zingwe, Keither R Abrams, Martin D Tobin, Louise Wain, Sue Carr, Edward Dove, Kamlesh Khunti, David Ford, Robert Free

**Affiliations:** 1 School of Psychology, Faculty of Health, Innovation, Technology and Science, Liverpool John Moores University, Liverpool, UK; 2 Department of Respiratory Sciences, University of Leicester, Maurice Shock Medical Sciences Building, University Road, Leicester, LE1 9HN UK; 3 Department of Infection and HIV Medicine, University Hospitals of Leicester NHS Trust, Leicester, UK; 4 Development Centre for Population Health, University of Leicester, Leicester, UK; 5 Faculty of Medical Sciences, University College London, London, UK; 6 College of Population Health, Health Sciences Centre, University of New Mexico, Albuquerque, USA; 7 Centre for Inequalities, Institute for Lifecourse Development, University of Greenwich, London, UK; 8 NIHR Leicester Biomedical Research Centre, Leicester, UK; 9 Division of Health Research, Lancaster University, Lancaster, UK

**Keywords:** Healthcare, Prospective cohort, Multilevel modelling, COVID-19, Alcohol

## Abstract

**Background:**

The COVID-19 pandemic exacerbated exposure to trauma and demands for healthcare workers (HCWs), which are known risks for heavy alcohol use and common mental disorders (CMD). We investigated the longitudinal associations between alcohol use and wider stressors with symptoms of CMD among HCWs.

**Methods:**

Data were obtained from the UK-REACH prospective cohort study of HCWs, collected between Dec 2020 and Feb 2021 (*N* = 12,821), and 6 months (*N* = 5164, 40% response rate) and 10 months later (*N* = 5454, 43% response rate). Symptoms of depression, anxiety, and post-traumatic stress disorder (PTSD), changes in frequency of alcohol use, COVID-19 stressors, occupational stressors, and discrimination were self-reported at each time point. Multilevel models analysed changes in symptoms of CMD over time and explored the associations with changes in alcohol use and wider stressors, for those who completed two or more surveys (*N* = 6973).

**Results:**

Mean symptoms of depression declined from baseline (1.07 ± 0.02) to 6-month (0.96 ± 0.02) and 10-month follow up (0.97 ± 0.02), as did mean symptoms of anxiety (baseline, 1.45 ± 0.02; 6-month, 1.35 ± 0.02; 10-month, 1.39 ± 0.02). Symptoms of PTSD only declined from baseline (3.36 ± 0.02) to 10-month follow-up (3.31 ± 0.02). More frequent alcohol use over time was associated with increased symptoms of depression (*β* = 0.31; 95% CI, 0.19 to 0.44), anxiety (*β* = 0.32; 95% CI, 0.18 to 0.45), and PTSD (*β* = 0.32; 95% CI, 0.18 to 0.46), as was bereavement due to COVID-19, and discrimination from patients and other staff. Occupational stressors were positively associated with symptoms of CMD, though this association was not as pronounced for those who drank less often (*β* = − 0.08; 95% CI, − 0.14 to − 0.02).

**Conclusions:**

We identified several mechanisms which contributed to worsened CMD, demonstrating that organisational changes are required to support HCWs to reduce their alcohol use, tackle discrimination, and to create a work environment where staff feel secure raising concerns.

**Supplementary Information:**

The online version contains supplementary material available at 10.1186/s12916-025-04474-4.

## Background

The COVID-19 pandemic negatively impacted mental health for many people [1, 2], particularly those with existing mental health problems [2]. Mental health problems often co-occur with at-risk alcohol use (i.e. drinking above recommended limits) [3], with alcohol sometimes being used to self-medicate and alleviate symptoms [4], or alternatively, heavy alcohol use can worsen mental health [5]. During the first lockdown in March 2020, it is estimated that between 25 and 50% of the general population in the United Kingdom (UK) increased their alcohol consumption, relative to before the pandemic [6], with the prevalence of at-risk alcohol use also rising during the first lockdown [7]. Individuals who were already drinking to at-risk levels and those with poor mental health were more likely to increase their consumption over time [8].

Prior to the COVID-19 pandemic, healthcare workers (HCWs) frequently experienced occupational strains, such as trauma exposure and interpersonal stressors [9]. These are known risk factors for common mental disorders (CMD), e.g. depression, anxiety, and post-traumatic stress disorder (PTSD), as well as maladaptive coping strategies, e.g. alcohol use [10]. The pandemic stretched the limits of healthcare systems and exacerbated exposure to trauma and demands for HCWs. The psychological impact of this has been well-documented with cross-sectional data, with women and nurses being consistently more likely to report poor mental health [11–14]. A global meta-review identified that the pooled prevalence of anxiety ranged from 16 to 41%, depression ranged from 14 to 27%, and post-traumatic stress disorder ranged from 18 to 56%, among HCWs during the pandemic [11]. Longitudinal studies of HCWs from various countries (not the UK) have shown mixed findings, with some indicating worsened mental health over the course of the pandemic, whereas others noted improvements in mental health [15].

Despite the known associations between alcohol use and mental health [3], and approximately 20% of HCWs drinking to at-risk levels (with a higher prevalence found in studies conducted during the COVID-19 pandemic) [16], no studies have assessed the associations between alcohol use and CMD among HCWs during the pandemic. We hypothesise that more frequent alcohol use will be associated with greater symptoms of CMD. We draw on Edmondson’s theory of psychological safety, whereby staff feel confident to voice concerns and that their organisation will act on these concerns [17], and the connection with physical safety (e.g. access to personal protective equipment; PPE) [18], to hypothesise that occupational stressors surrounding psychological and physical safety will be associated with increased symptoms of CMD. Building on existing evidence, we also hypothesise that COVID-19 related stressors (e.g. previous infection and bereavement) and workplace discrimination will be associated with increased symptoms of CMD [19–21]. Identifying the mechanisms which contribute to worsened mental health among HCWs is of vital public health importance to ensure a healthy workforce and a resilient post-pandemic recovery.

Using longitudinal data from the national UK Research study into Ethnicity and COVID-19 outcomes in Healthcare workers (UK-REACH) [22, 23], we investigated changes in self-reported symptoms of CMD, at three time-points during the pandemic. We examined whether more frequent alcohol use over time, COVID-19 related-stressors, occupational stressors, and discrimination, were associated with increased symptoms of CMD. We also explored the interaction between occupational stressors and changes in frequency of alcohol use with symptoms of CMD, hypothesising that the association between more frequent alcohol use and increased symptoms of CMD will be stronger for those who experienced greater occupational stressors. This builds on previous UK-REACH qualitative work, exploring the lived experience of HCWs from diverse ethnic backgrounds during the pandemic, and factors that had an impact on their mental health [24].

## Methods

### Study design

This analysis used data from the UK-REACH prospective cohort, which is part of a larger programme of research that was established to investigate the impact of the COVID-19 pandemic on HCWs and to explore differences across ethnic groups [22, 23]. UK healthcare and ancillary workers aged 16 or over were invited to participate via a hyperlink distributed by healthcare professional regulators, or directly through participating healthcare trusts and advertisements on social media and in newsletters. Interested participants were directed to the cohort website, where they could read the participant information sheet and provide informed electronic consent. Participants completed the online baseline questionnaire between 4th December 2020 and 28th February 2021 (during the second wave of the COVID-19 pandemic, a third national lockdown, and the beginning of the vaccination programme). Consenting participants were asked to complete follow-up online questionnaires 6 months (21st April 2021–26th June 2021; most people had been offered the first dose of the vaccine and restrictions were lifted) and 10 months (18th October 2021–26th November 2021; third wave of infections and rise in hospitalisations and deaths) after the study opened. The questionnaire included topics relating to demographics; ethnicity, nationality, religion, and languages; work; home and social life; harassment and discrimination; physical and mental health; COVID-19 experiences and beliefs; and psychological measures [22]. Participants who completed each survey were entered into a prize draw to win gift vouchers.

### Study population

Of 17,891 individuals recruited to UK-REACH, 15,119 individuals responded to the baseline questionnaire (response rate = 84.5%). A total of 5632 participants completed the 6-month follow-up (response rate = 31.4% of consenting participants) and 6535 completed the 10-month follow-up (response rate = 36.5%). Each questionnaire was designed so that it could either be standalone or used in a longitudinal arrangement, meaning participants could complete a single follow-up questionnaire without completing others. The analytical sample was restricted to those who completed at least two surveys (i.e. baseline and at least one follow-up survey). Comparisons of the demographic characteristics of the cohort at baseline with the target population are reported elsewhere [22]. To summarise, the UK-REACH cohort has a similar age and sex distribution to the NHS workforce, but is more ethnically diverse, with 27% of the UK-REACH cohort reporting ethnic minority status, compared to 24% of the NHS workforce [22].

### Measures of symptoms of CMD

In each questionnaire, symptoms of anxiety, depression, and PTSD were measured using the following screening instruments: 2-item Generalised Anxiety Disorder (GAD-2) scale [25], 2-item Patient Health Questionnaire (PHQ-2) [26], 2-item PTSD checklist-civilian version (PCL-C) [27]. For the GAD-2 and PHQ-2, responses to each item ranged from ‘not at all’ to ‘nearly every day’ (scores ranged from zero to six). Responses to each item of the PCL-C were on a 5-point Likert scale, ranging from ‘not at all’ to ‘extremely’ (scores ranged from two to 10).

### Measures of alcohol consumption

In the baseline questionnaire, frequency of alcohol use was determined by asking participants how often they have a drink containing alcohol (never; monthly or less; two to four times a month; two to three times per week; four or more times a week), with responses recoded to reflect three levels of frequency to increase statistical power: never, less than four times a week, and four or more times a week. Participants who reported drinking more than monthly were asked how many units they drink in a typical week (1–7, 8–14, 15–21, 22–28, 29–35, 36–50, 51 + units). At-risk alcohol use at baseline was determined using information from these variables: low risk drinkers (≤ 14 units; including those who reported drinking monthly or less) *versus* at-risk drinkers (> 14 units, according to UK government guidelines) [28].

Each survey measured changes in frequency of alcohol use since the pandemic began (baseline questionnaire), in the past 4 months (6-month follow-up) and in the past 6 months (10-month follow-up). Responses included the following: never drank, has not changed, drink less often, drink more often.

### COVID-19 related-factors, occupational stressors, and discrimination

Across each survey, participants were asked if they had ever had a test for COVID-19 (either swab test for active infection or antibody test for previous infection) and if so, whether they had ever had a positive test result [29]. Bereavement was measured through a single item that asked if participants knew anyone who had died from COVID-19 (not including patients).

At each survey, participants were asked if they had appropriate access to personal protective equipment (PPE) at work, with the following responses: all/most of the time, some of the time, rarely/not at all. Participants were asked the extent to which they agree that they would feel secure raising concerns about unsafe clinical practice and that they would be confident that their organisation would address their concern: strongly agree/agree, neither agree nor disagree, strongly disagree/disagree. Participants only responded to these measures if they reported working at the time. Responses to these items were summed to create a score ranging from 0 to 9.

Across all surveys, experiences of discrimination at work were measured with a three-level categorical variable: no experiences of discrimination, discrimination from patients/public, discrimination from other staff.

### Demographic and occupational variables (covariates)

At baseline, participants reported their age, gender, marital status, and highest level of educational attainment. Participants were asked to select their ethnic group from 18 categories and asked whether they were born in the UK or elsewhere. Healthcare role, current working status (working/not working) and working status at the start of the first lockdown, were obtained from the baseline survey. To reduce the likelihood of biased estimates, we did not include smoking, physical health diagnoses, or health-related quality of life, as covariates. Physical health diagnoses and health-related quality of life can be colliders (caused by both the exposure and the outcome), resulting in collider bias, and smoking can mediate the association between the exposure and the outcome, resulting in overadjustment bias.

### Statistical analysis

Frequencies (and percentages) for the demographic characteristics of the sample, frequency of alcohol use, and at-risk alcohol use were estimated from the baseline survey. Mean symptom scores (with standard deviations) for each measure of CMD and frequencies (and percentages) for changes in frequency of alcohol use were estimated at each time point.

Due to the hierarchical structure of the data (repeated measurements nested within individuals), multi-level linear regression models (MLM) were conducted to analyse changes in symptoms of CMD, analysing each outcome measure separately, with timepoint as a categorical exposure. MLM partitions the overall variance in outcomes into separate levels, determining predictors of within and between participant variances. Two-level random intercept, fixed slope models were tested.

Predictors were included in steps. First, baseline frequency of alcohol consumption (level two predictor: vary by participant) and changes in frequency of alcohol use (level one predictor: vary by time point) were added to the model. Second, COVID-19 infection and bereavement were added (level one predictors). Then, occupational stressors and discrimination were included as level one predictors, restricting these analyses to those who were working at the time of completing the survey. Finally, the interaction between psychological stressors relating to work and changes in frequency of alcohol use were added to the model.

The analyses were conducted in STATA SE 15·1, using the *mixed* command to conduct MLMs. Demographic and occupational variables were included as covariates across all MLMs. Beta coefficients (β) with standard errors are reported. Mean symptoms of CMD at each time point for each sub-group of the explanatory variables are reported using the *margins* command. The intraclass correlation coefficient (ICC, the association between observations within individuals), Akaike Information Criteria (AIC) and Bayes Information Criteria (BIC), are reported for each step.

### Missing data

The analytical sample was restricted to those who completed at least two surveys. An inverse probability weight was created to determine predictors of attrition from the study. Regression models were conducted to identify variables which were significant predictors of both attrition (*versus* responding to all three surveys; Table S1, Additional File 1) and symptoms of mental health (Table S2, S3 and S4; Additional File 1), which were used to create inverse probability weights (Table S5; Additional File 1). The inverse probability weight was applied when running the MLMs, using the *pweight* command. Missing data for all variables across the surveys were minimal, with less than 5% missing for each variable (including ‘prefer not to say’ responses which were recoded as missing, Table S6; Additional File 1).

### Ethical approval

The UK-REACH study was approved by the Health Research Authority (Brighton and Sussex Research Ethics Committee: 20/HRA/4718). All participants provided written, informed, consent.

### Patient and public involvement

A Stakeholder Advisory Group, including representatives from national and local organisations, and a Professional Expert Panel of healthcare workers from varied ethnic backgrounds, genders, and occupations, were involved in formulating research questions and designing data collection methods for UK-REACH.

## Results

### Sample characteristics

The participant flow diagram is outlined in Figure S1 (Additional File 2). In total, 12,821 participants completed at least one measure of mental health at baseline (6-month follow up *N* = 5164; 10-month follow-up *N* = 5454), with 5848 participants excluded as they only completed the baseline survey. The final analytical sample included 6973 individuals (*N* = 3645 completed all three time points; *N* = 1519 responded to the baseline and 6-month follow-up surveys; *N* = 1809 responded to the baseline and 10-month follow-up surveys). The demographic characteristics of the analytical sample are presented in Table 1.

**Table 1:** Demographic characteristics of the analytical sample at baseline (*N* = 6973). Percentages are weighted to account for attrition

		**Baseline**
		***N* (%)**
Age (years)	16–35	1771 (25.71)
	36–45	1654 (23.78)
	46–55	1730 (24.72)
	> 55	1818 (25.79)
Gender	Man	1694 (24.20)
	Woman	5268 (75.63)
	I use another term	9 (0.14)
	Prefer not to say	1 (0.01)
	*Missing*	*1 (0.01)*
Marital status	Single	1104 (15.98)
	Living with partner	1031 (14.82)
	Married	4131 (59.16)
	Divorced/separated	522 (7.46)
	Widowed	69 (0.98)
	Prefer not to say	29 (0.43)
	*Missing*	*87 (1.16)*
Educational attainment	A-level or below	1087 (15.65)
	Undergraduate degree	3273 (47.15)
	Postgraduate degree	2592 (36.91)
	Prefer not to say	7 (0.10)
	*Missing*	*14 (0.19)*
Ethnic group	White British	4402 (62.81)
	Any other White background	671 (9.57)
	Indian	609 (8.92)
	Pakistani and Bangladeshi	197 (2.88)
	Any other Asian background	354 (5.13)
	Black	240 (3.53)
	Mixed	282 (4.07)
	Any other ethnic group	124 (1.82)
	Prefer not to say	3 (0.04)
	*Missing*	*91 (1.21)*
Country of birth	UK	5314 (75.89)
	Elsewhere	1644 (23.90)
	Prefer not to say	4 (0.06)
	*Missing*	*11 (0.15)*
Job role	Medical/medical support	1675 (23.98)
	Nursing	1455 (20.87)
	Allied health professionals	2859 (41.14)
	Dental	380 (5.57)
	Administrative/other	391 (5.59)
	Prefer not to say	4 (0.05)
	*Missing*	*209 (2.79)*

### Changes in symptoms of CMD

The frequency of alcohol use at baseline, changes in the frequency of alcohol use over time, at-risk alcohol use at baseline, and mean symptoms of CMD are summarised in Table S7 (see Table S8 for results restricted to those who completed all three surveys; Additional File 1).

Null models with no random intercept were first estimated to examine mean symptoms of depression, anxiety, and PTSD, before adding a random intercept to the model (accounting for repeated measures in participants). The AIC and BIC were lower for the random intercept models compared to the null models, indicating that MLMs are a better fit to the data (Table 2). The ICC indicated that 55% of the variance in symptoms of depression, 57% of the variance in symptoms of anxiety, and 60% of the variance in symptoms of PTSD were at the participant level.

**Table 2:** Comparison of the null model and random intercept model

	**Depression**	**Anxiety**	**PTSD**
Null model			
* N* observations	17,354	17,449	17,497
* β*	1.00	1.40	3.32
95% CI	0.97 to 1.02	1.37 to 1.42	3.29 to 3.35
AIC	62,318.33	66,656.26	70,670.90
BIC	62,326.09	66,664.03	70,678.67
Random intercept			
* N* Groups	6894	6927	6947
* β*	1.01	1.40	3.34
95% CI	0.98 to 1.04	1.37 to 1.44	3.30 to 3.37
AIC	51,711.37	55,074.35	58,309.61
BIC	51,734.65	55,097.65	58,332.92
ICC	0.55	0.57	0.60

Compared to mean symptoms of depression at baseline (1.07 ± 0.02), symptoms of depression significantly decreased at 6-month follow up (0.96 ± 0.02; *β* = − 0.11; 95% confidence interval (CI), − 0.14 to − 0.07) and at 10-month follow up (0.97 ± 0.02; *β* = − 0.10; 95% CI, − 0.14 to − 0.07). Similarly, mean symptoms of anxiety also decreased significantly from baseline (1.45 ± 0.02) to 6-month (1.35 ± 0.02; *β* = − 0.10; 95% CI, − 0.13 to − 0.06) and 10-month follow-up (1.39 ± 0.02; *β* = − 0.06; 95% CI, − 0.10 to − 0.02). Symptoms of PTSD did not significantly decrease from baseline (3.36 ± 0.02) to 6-month follow-up (3.33 ± 0.02; *β* = − 0.03; 95% CI, − 0.07 to 0.01) but did decrease from baseline to 10-month follow-up (3.31 ± 0.02; *β* = − 0.05; 95% CI, − 0.09 to − 0.01). However, the changes in mean symptoms represent small effects.

### Mechanisms associated with changes in CMD

The results of the MLMs for depression, anxiety, and PTSD, are reported in Tables 3, 4, and 5, respectively. Frequency of alcohol use at baseline and change in frequency of alcohol use over time explained 4% of the variance in symptoms of depression (ICC = 0.51), 3% of the variance in symptoms of anxiety (ICC = 0.54), and 2% of the variance in symptoms of PTSD (ICC = 0.58). Those who reported drinking more frequently over time, compared to those who have never drank alcohol, reported increased symptoms of depression (*β* = 0.31; 95% CI, 0.19–0.44); anxiety (*β* = 0.32; 95% CI, 0.18–0.45); and PTSD (*β* = 0.32; 95% CI, 0.18–0.46), representing medium effect sizes.

**Table 3:** Multilevel modelling analyses including level one predictors (vary by time point) and level two predictors (vary by participant) of symptoms of depression. Analyses are weighted to account for attrition and adjusted for demographic and occupational variables (age, gender, marital status, educational attainment, ethnicity, country of birth, healthcare role)

			**Marginal means**		
	**β**	**95% CI**	**Baseline**	**6 month**	**10 month**
**Step 1 ICC = 0.51**	*AIC* = *48,006.25*	*BIC* = *48,252.64*	*N* obs = 16,314	*N* groups = 6521	
Frequency of alcohol use					
Never (reference)	0.00		1.09	1.00	1.03
< 4 times a week	− 0.08	− 0.21 to 0.06	1.02	0.93	0.95
4 + times a week	− 0.02	− 0.18 to 0.15	1.08	0.98	1.01
Change in alcohol use					
Never drank (reference)	0.00		0.98	0.89	0.92
Has not changed	− 0.02	− 0.13 to 0.09	0.96	0.87	0.90
Drink less often	− 0.09	− 0.03 to 0.21	1.07	0.99	1.01
Drink more often	0.31***	0.19 to 0.44	1.29	1.20	1.23
**Step 2 ICC = 0.51**	*AIC* = *47,913.86*	*BIC* = *48,183.30*	*N* obs = 16,292	*N* groups = 6521	
Previous infection					
No (reference)	0.00		1.03	0.94	0.96
Yes	0.02	− 0.03 to 0.08	1.05	0.96	0.98
Unsure	0.09	− 0.01 to 0.18	1.11	1.02	1.05
Bereavement					
No (reference)	0.00		0.98	0.89	0.91
Yes	0.13***	0.09 to 0.17	1.11	1.02	1.04
**Step 3**^**a**^** ICC = 0.48**	*AIC* = *44,733.71*	*BIC* = *45,023.91*	*N* obs = 15,136	*N* groups = 6458	
Work stressors (continuous)	0.07***	0.05 to 0.09	/	/	/
Discrimination at work					
No discrimination (reference)	0.00		0.95	0.88	0.87
From patients/public	0.20***	0.12 to 0.27	1.14	1.08	1.07
From other staff	0.52***	0.44 to 0.61	1.47	1.40	1.39
**Step 4**^**a**^** ICC = 0.48**	*AIC* = *44,728.23*	*BIC* = *45,041.34*	*N* obs = 15,136	*N* groups = 6458	
Work stressors # change in alcohol use					
Work stressors # never (reference)	0.00		/	/	/
Work stressors # has not changed	− 0.02	− 0.06 to 0.03	/	/	/
Work stressors # drink less often	− 0.08*	− 0.14 to − 0.02	/	/	/
Work stressors # drink more often	− 0.01	− 0.07 to 0.05	/	/	/

^*^p < 0.05, **p < 0.01, ***p < 0.001, ^a^Analyses are restricted to those working at the time

**Table 4:** Multilevel modelling analyses including level one predictors (vary by time point) and level two predictors (vary by participant) of symptoms of anxiety. Analyses are weighted to account for attrition and adjusted for demographic and occupational variables (age, gender, marital status, educational attainment, ethnicity, country of birth, healthcare role)

				**Marginal means**		
		**β**	**95% CI**	**Baseline**	**6 month**	**10 month**
**Step 1 ICC = 0.54**		*AIC* = *51,386.77*	*BIC* = *51,633.34*	*N* obs = 16,402	*N* groups = 6551	
Frequency of alcohol use						
Never (reference)		0.00		1.50	1.43	1.48
< 4 times a week		− 0.11	− 0.26 to 0.04	1.39	1.31	1.37
4 + times a week		− 0.01	− 0.18 to 0.17	1.50	1.42	1.47
Change in alcohol use						
Never drank (reference)		0.00		1.34	1.26	1.31
Has not changed		0.03	− 0.08 to 0.15	1.37	1.29	1.34
Drink less often		0.11	− 0.01 to 0.24	1.45	1.37	1.42
Drink more often		0.32***	0.18 to 0.45	1.65	1.57	1.62
**Step 2 ICC = 0.54**		*AIC* = *51,292.13*	*BIC* = *51,561.76*	*N* obs = 16,381	*N* groups = 6551	
Previous infection						
No (reference)		0.00		1.41	1.34	1.39
Yes		− 0.01	− 0.07 to 0.05	1.54	1.46	1.51
Unsure		0.12*	0.02 to 0.22	1.41	1.33	1.38
Bereavement						
No (reference)		0.00		1.36	1.29	1.34
Yes		0.13***	0.08 to 0.18	1.49	1.42	1.47
**Step 3**^**a**^** ICC = 0.51**		*AIC* = *47,942.86*	*BIC* = *48,233.26*	*N* obs = 15,401	*N* groups = 6487	
Work stressors (continuous)		0.09***	0.06 to 0.11	/	/	/
Discrimination at work						
No discrimination (reference)		0.00		1.32	1.28	1.29
From patients/public		0.24***	0.15 to 0.32	1.55	1.51	1.53
From other staff		0.56***	0.46 to 0.65	1.87	1.83	1.85
**Step 4**^**a**^** ICC = 0.51**		*AIC* = *47,945.98*	*BIC* = *48,259.31*	N obs = 15,401	N groups = 6487	
Work stressors # change in alcohol use						
Work stressors # never (reference)		0.00		/	/	/
Work stressors # has not changed		0.02	− 0.03 to 0.07	/	/	/
Work stressors # drink less often		− 0.01	− 0.07 to 0.05	/	/	/
Work stressors # drink more often		0.03	− 0.04 to 0.10	/	/	/

^*^p < 0.05, **p < 0.01, ***p < 0.001, ^a^Analyses are restricted to those working at the time

**Table 5:** Multilevel modelling analyses including level one predictors (vary by time point) and level two predictors (vary by participant) of symptoms of PTSD. Analyses are weighted to account for attrition and adjusted for demographic and occupational variables (age, gender, marital status, educational attainment, ethnicity, country of birth, healthcare role)

			**Marginal means**		
	**β**	**95% CI**	**Baseline**	**6 month**	**10 month**
**Step 1 ICC = 0.58**	*AIC* = *54,404.45*	*BIC* = *54,651.1*	*N* obs = 16,447	*N* groups = 6571	
Frequency of alcohol use					
Never (reference)	0.00		3.44	3.42	3.42
< 4 times a week	− 0.16	− 0.32 to 0.01	3.28	3.27	3.27
4 + times a week	− 0.09	− 0.29 to 0.10	3.34	3.34	3.33
Change in alcohol use					
Never drank (reference)	0.00		3.24	3.23	3.22
Has not changed	0.02	− 0.10 to 0.14	3.26	3.25	3.25
Drink less often	0.07	− 0.05 to 0.20	3.31	3.30	3.30
Drink more often	0.32***	0.18 to 0.46	3.56	3.55	3.54
**Step 2 ICC = 0.57**	*AIC* = *54,259.99*	*BIC* = *54,529.73*	*N* obs = 16,426	*N* groups = 6571	
Previous infection					
No (reference)	0.00		3.28	3.28	3.27
Yes	0.07*	0.00 to 0.14	3.48	3.48	3.46
Unsure	0.20**	0.08 to 0.31	3.36	3.35	3.34
Bereavement					
No (reference)	0.00		3.21	3.21	3.19
Yes	0.23***	0.17 to 0.28	3.44	3.43	3.42
**Step 3**^**a**^** ICC = 0.55**	*AIC* = *50,633.38*	*BIC* = *50,923.90*	*N* obs = 15,448	*N* groups = 6508	
Work stressors (continuous)	0.08***	0.05 to 0.10	/	/	/
Discrimination at work					
No discrimination (reference)	0.00		3.18	3.21	3.14
From patients/public	0.32***	0.23 to 0.41	3.50	3.53	3.46
From other staff	0.69***	0.58 to 0.80	3.87	3.90	3.83
**Step 4**^**a**^** ICC = 0.55**	*AIC* = *50,639.20*	*BIC* = *50,952.66*	*N* obs = 15,448	*N* groups = 6508	
Work stressors # change in alcohol use					
Work stressors # never (reference)	0.00		/	/	/
Work stressors # has not changed	0.00	− 0.06 to 0.06	/	/	/
Work stressors # drink less often	− 0.00	− 0.07 to 0.07	/	/	/
Work stressors # drink more often	− 0.01	− 0.07 to 0.09	/	/	/

^*^p < 0.05, **p < 0.01, ***p < 0.001, ^a^Analyses are restricted to those working at the time

Bereavement due to COVID-19, but not infection, was associated with higher symptoms of depression (*β* = 0.13; 95% CI, 0.09–0.17) and anxiety (*β* = 0.13; 95% CI, 0.08–0.18). However, COVID-19 infection was associated with greater symptoms of PTSD (*β* = 0.07; 95% CI, 0.00–0.14), as was bereavement (*β* = 0.23; 95% CI, 0.17–0.28), though effect sizes were small.

Occupational stressors and discrimination explained a further 3% of the variance depression (ICC = 0.48) and anxiety (ICC = 0.51), and 2% of the variance in symptoms of PTSD (ICC = 0.55). Greater occupational stressors being associated with greater symptoms of depression (*β* = 0.07; 95% CI, 0.05–0.09); anxiety (*β* = 0.09; 95% CI, 0.06–0.11); and PTSD (*β* = 0.23; 95% CI, 0.17–0.28), with small effect sizes. Discrimination from patients/public was associated with increased symptoms of depression (*β* = 0.20; 95% CI, 0.12–0.27); anxiety (*β* = 0.24; 95% CI, 0.15–0.32); and PTSD (*β* = 0.32; 95% CI, 0.23–0.41), as was discrimination from other staff (depression, *β* = 0.52; 95% CI, 0.44–0.61; anxiety, *β* = 0.52; 95% CI, 0.46–0.65; PTSD, *β* = 0.69; 95% CI, 0.58–0.80), with the latter representing large effects.

### Interaction between occupational stressors and alcohol use

There was no significant interaction between occupational stressors and changes in frequent alcohol use with symptoms of anxiety or PTSD. There was a significant negative interaction between occupational stressors and drinking less often, with symptoms of depression (*β* = − 0.08; 95% CI, − 0.14 to − 0.02). This indicates that the effect of occupational stressors on symptoms of depression was not as strong for those who reported less frequent alcohol use over time. However, the effect size was small.

## Discussion

In one of the only longitudinal studies of UK HCWs, we examined changes in symptoms of CMD at three time-points during the COVID-19 pandemic and investigated whether changes in alcohol use, COVID-19-related stressors, occupational stressors, and discrimination were associated with worsened symptoms of CMD. Though there was a statistically significant decline in symptoms of depression, anxiety, and PTSD from baseline to 6- and/or 10-month follow-up, the effects were small, and there were no differences between mean symptoms from 6-month to 10-month follow-up. We identified several mechanisms which were associated with increased symptoms of CMD. HCWs who drank more often reported greater symptoms of CMD, as did those who had lost someone due to COVID-19. In addition, occupational stressors and discrimination from patients/public and other staff were related to greater symptoms of CMD. We identified an interaction between alcohol use and occupational stressors with symptoms of depression, whereby the effect of occupational stressors on symptoms of depression were not as pronounced for people who drank less often over time.

There is a growing body of evidence to suggest that HCWs experienced poor mental health outcomes during the COVID-19 pandemic [11, 12, 30]. Cross-sectional studies have identified that over half of UK HCWs met criteria for a CMD during the COVID-19 pandemic [31]. These studies were conducted in the first few months of the COVID-19 pandemic and first government-mandated lockdown [13], or within the first year [31]. Our study is one of the only longitudinal studies of UK HCWs, showing that symptoms of CMD slightly declined in spring 2021, when widespread vaccination had occurred and restrictions were lifted, compared to responses during the second wave of infections (baseline survey, which also coincided with a third national lockdown). Symptoms of CMD did not change from spring 2021 to the third wave of infections (10-month follow-up), despite hospitalisations and deaths rising. Global longitudinal evidence on changes in mental health among HCWs is mixed, with some studies identifying negative changes in mental health, whereas others found improvements in mental health over time [15]. The collated evidence outlines that a considerable proportion of HCWs in the UK experienced poor mental health during the COVID-19 pandemic, and as pressure on the NHS remains high, we must ensure mental health support is available and accessible.

Our analysis showed positive associations between increased frequency of alcohol use and symptoms of CMD, suggesting that some HCWs may have drank more often to cope with worsened mental health, aligning with wider research conducted during the COVID-19 pandemic [32–34]. Increased frequency of alcohol use was associated with greater symptoms of CMD, and the positive association between occupational stressors and depression symptoms was not as pronounced for HCWs who drank less often. We also identified wider mechanisms which were associated with worsened mental health. HCWs working on the frontline during the pandemic often witnessed patients dying from COVID-19, as well as the disruptions to grieving that their families and loved ones experienced [35]. Yet, experiencing their own loss due to COVID-19 likely contributed to a layering of distress, and these complex emotions can result in poor mental health [36]. Concerningly, we found that HCWs who have experienced discrimination from patients/public and/or other staff reported much higher symptoms of CMD. This aligns with quantitative research showing that discrimination was associated with greater symptoms of depression among HCWs during the COVID-19 pandemic [21], with previous qualitative work also indicating that discrimination is a central challenge for minoritised HCWs [37].

The NHS is experiencing severe pressures, with the pandemic exacerbating existing strains due to years of under-resourcing. To ensure a healthy workforce, it is critical that policies are implemented to support the NHS, particularly as approximately 20% of HCWs are actively seeking employment outside of the NHS [38]. Occupational stressors, including a lack of physical and psychological safety at work, were strongly associated with symptoms of CMD, suggesting that policies enabling equitable and immediate access to PPE could have reduced symptoms of CMD, and this must be considered in future pandemic preparedness. Organisations must strive to build psychologically safe places, where staff feel secure in raising concerns and feel confident that their concerns will be addressed. Workplace discrimination is a major concern within the NHS, having detrimental impacts on mental health [39, 40]. Structural and institutional changes are required, including but not limited to widespread anti-racism training, increasing accountability of leaders, ensuring a diverse workforce with diverse leadership teams, and creating safe spaces where staff can speak about their experiences.

## Strengths and limitations

This is the first longitudinal analysis of the associations between alcohol use, occupational stressors, and symptoms of CMD among UK HCWs during the COVID-19 pandemic. This analysis has several strengths, including the large sample size and length of follow up. An inverse probability weight was created to account for attrition, and non-response to individual survey items was minimal. Additionally, the response rates for UK-REACH were greater than other longitudinal surveys of HCWs conducted during the pandemic, e.g. [30]. However, this analysis has limitations. Primarily, the quantity of alcohol consumed was not measured in follow-up surveys and some participants may have increased the frequency of their alcohol use without increasing the quantity. In addition, the UK-REACH survey did not include measures of mental health prior to COVID-19, therefore the extent to which mental health worsened as a result of the pandemic is not known. As this was a secondary analysis, there may be other confounders that were not accounted for, such as substance use and medications used to treat mental health, because they were not available in the survey. Further, though the multilevel model accounts for intra-group correlation, some groups may be more homogenous than others, meaning the within-group variance could be quite low for some individuals, reducing the power to detect time-varying effects. Finally, though the UK-REACH sample is demographically similar to the NHS workforce in terms of age and gender, the cohort includes a large proportion of staff from ethnic minority backgrounds [22]. This impacts on our ability to make generalisations to the wider NHS workforce, as there may be ethnic differences in CMD and alcohol use, as well as in experiences of wider stressors.

## Conclusions

Among UK HCWs, symptoms of CMD declined slightly from the baseline survey, during the second wave of COVID-19 infections and a third national lockdown, to spring 2021, when widespread vaccination had occurred and restrictions were lifted. HCWs who reported drinking alcohol more frequently showed greater increases in symptoms of CMD than those who did not drink alcohol. Occupational stressors (e.g. inconsistent access to PPE) and discrimination at work were also strongly associated with greater symptoms of CMD. The effect of occupational stressors on symptoms of depression was not as strong for those who reduced the frequency of their alcohol use. These findings suggest that whilst interventions focused solely on reducing alcohol use would be beneficial, complementary psychological support is also needed to improve mental health.

## Supplementary Information

Additional file 1. Tables S1–S8. Table S1. Regression analyses of baseline variables associated with attrition (completing 1 or 2 surveys versus completing all 3 surveys) (N = 11,508). Table S2. Regression analyses of baseline variables associated with depression (N = 11,515). Table S3. Regression analyses of baseline variables associated with anxiety (N = 11,515). Table S4. Regression analyses of baseline variables associated with PTSD (N = 11,515). Table S5. Final regression model of baseline variables associated with both attrition and mental health (N = 11,695). Table S6. Descriptive statistics and missing data for level one predictor variables (vary by time point) at each time point, for participants who completed at least two surveys (N = 6973). Percentages are weighted. Table S7. Characteristics of alcohol use and mental health at each time point. Percentages are weighted to account for attrition at follow up. Table S8. Prevalence of alcohol use and mental health at each time point for those with completed all three surveys (N = 3645). Percentages are weighted to account for attrition at follow up

Additional file 2. Figure S1. Figure S1. Participant flow diagram

Additional file 3.

## Data Availability

To access data or samples produced by the UK-REACH study, the working group representative must first submit a request to the Core Management Group by contacting the UK-REACH Project Manager in the first instance. For ancillary studies outside of the core deliverables, the Steering Committee will make final decisions once they have been approved by the Core Management Group. Decisions on granting the access to data/materials will be made within eight weeks. Third party requests from outside the Project will require explicit approval of the Steering Committee once approved by the Core Management Group. Note that should there be significant numbers of requests to access data and/or samples then a separate Data Access Committee will be convened to appraise requests in the first instance.

## Abbreviations

UK: United Kingdom
HCWs: Healthcare workers
PTSD: Post-traumatic stress disorder
CMD: Common mental disorders
UK-REACH: UK Research study into Ethnicity and COVID-19 outcomes in Healthcare workers
GAD: Generalised anxiety disorder
PHQ: Patient health questionnaire
PCL-C: PTSD checklist–civilian version
PPE: Personal protective equipment
MLM: Multi-level models
ICC: Intraclass correlation coefficient
AIC: Akaike Information Criteria
BIC: Bayes Information Criteria
CI: Confidence interval
