# Supplementary material for: Longitudinal associations between alcohol use, occupational stressors, and mental health among healthcare and ancillary workers in the United Kingdom during the COVID-19 pandemic (UK-REACH)
Source: BMC Med. 2025 Nov 28;23:665. doi: 10.1186/s12916-025-04474-4 (PMC12664230; doi:10.1186/s12916-025-04474-4)
Supplement: Supplementary file 1 — Additional file 1. Tables S1–S8. Table S1. Regression analyses of baseline variables associated with attrition (completing 1 or 2 surveys versus completing all 3 surveys) (N = 11,508). Table S2. Regression analyses of baseline variables associated with depression (N = 11,515). Table S3. Regression analyses of baseline variables associated with anxiety (N = 11,515). Table S4. Regression analyses of baseline variables associated with PTSD (N = 11,515). Table S5. Final regression model of baseline variables associated with both attrition and mental health (N = 11,695). Table S6. Descriptive statistics and missing data for level one predictor variables (vary by time point) at each time point, for participants who completed at least two surveys (N = 6973). Percentages are weighted. Table S7. Characteristics of alcohol use and mental health at each time point. Percentages are weighted to account for attrition at follow up. Table S8. Prevalence of alcohol use and mental health at each time point for those with completed all three surveys (N = 3645). Percentages are weighted to account for attrition at follow up [file 12916_2025_4474_MOESM1_ESM.docx]

Supplementary Materials

[**Table S1. Regression analyses of baseline variables associated with attrition (completing 1 or 2 surveys *versus* completing all 3 surveys) (N = 11,508)** 2](#_Toc211863214)

[**Table S2. Regression analyses of baseline variables associated with depression (N = 11,515)** 3](#_Toc211863215)

[**Table S3. Regression analyses of baseline variables associated with anxiety (N = 11,515)** 4](#_Toc211863216)

[**Table S4. Regression analyses of baseline variables associated with PTSD (N = 11,515)** 5](#_Toc211863217)

[**Table S5. Final regression model of baseline variables associated with both attrition and mental health (N = 11,695)** 6](#_Toc211863218)

[**Table S6. Descriptive statistics and missing data for level one predictor variables (vary by time point) at each time point, for participants who completed at least two surveys (N = 6,973). Percentages are weighted.** 7](#_Toc211863219)

[**Table S7. Characteristics of alcohol use and mental health at each time point. Percentages are weighted to account for attrition at follow up.** 8](#_Toc211863220)

[**Table S8. Prevalence of alcohol use and mental health at each time point for those with completed all three surveys (N = 3,645). Percentages are weighted to account for attrition at follow up.** 9](#_Toc211863221)

# **Table S1. Regression analyses of baseline variables associated with attrition (completing 1 or 2 surveys *versus* completing all 3 surveys) (N = 11,508)**

|  |  | **Attrition** |  |
| --- | --- | --- | --- |
|  |  | **OR** | **95% CI** |
| Mental health | Depression | 1.06** | 1.02 to 1.10 |
|  | Anxiety | 0.96* | 0.92 to 0.99 |
|  | PTSD | 1.05** | 1.02 to 1.08 |
| Frequency of alcohol use | Never drank | 1.00 |  |
|  | Less than 4x a week | 1.23** | 1.06 to 1.44 |
|  | 4+ times a week | 1.13 | 0.93 to 1.39 |
| At-risk alcohol use | Non-drinkers/low risk | 1.00 |  |
|  | At-risk drinkers | 1.12 | 0.96 to 1.31 |
| COVID-19 infection | No | 1.00 |  |
|  | Yes | 1.17** | 1.05 to 1.30 |
|  | Unsure | 1.23* | 1.04 to 1.46 |
| Bereavement | No | 1.00 |  |
|  | Yes | 1.13** | 1.03 to 1.23 |
| Work stressors | Work stressors | 1.02 | 0.99 to 1.05 |
| Discrimination | No discrimination | 1.00 |  |
|  | From patients/public | 0.93 | 0.82 to 1.05 |
|  | From other staff | 1.12 | 0.98 to 1.28 |
| Gender | Male | 1.00 |  |
|  | Female | 1.16** | 1.05 to 1.28 |
| Age | Age >35 | 1.00 |  |
|  | Age 36-45 | 0.86* | 0.76 to 0.98 |
|  | Age 46-55 | 0.79*** | 0.69 to 0.90 |
|  | Age 55+ | 0.69*** | 0.60 to 0.78 |
| Marital status | Single | 1.00 |  |
|  | Living with partner | 1.19* | 1.02 to 1.37 |
|  | Married | 1.08 | 0.95 to 1.22 |
|  | Divorced/separated | 1.25* | 1.03 to 1.51 |
|  | Widowed | 1.25 | 0.80 to 1.94 |
| Education | A-Level or below | 1.00 |  |
|  | Undergraduate degree | 0.92 | 0.81 to 1.05 |
|  | Postgraduate degree | 0.83** | 0.72 to 0.94 |
| Ethnicity | White British | 1.00 |  |
|  | Any other White background | 0.86 | 0.73 to 1.02 |
|  | Indian | 1.41*** | 1.18 to 0.68 |
|  | Pakistani & Bangladeshi | 1.37* | 1.04 to 1.80 |
|  | Any other Asian background | 0.92 | 0.75 to 1.13 |
|  | Black | 1.34* | 1.05 to 1.72 |
|  | Mixed | 1.12 | 0.90 to 1.38 |
|  | Any other ethnic group | 1.66** | 1.16 to 2.37 |
| Country of birth | UK born | 1.00 |  |
|  | Born elsewhere | 1.44*** | 1.26 to 1.65 |
| Job role | Medical role | 1.00 |  |
|  | Nursing | 1.18* | 1.03 to 1.36 |
|  | Allied health professional | 1.32*** | 1.18 to 1.47 |
|  | Dental | 1.34** | 1.10 to 1.62 |
|  | Administrative | 1.24* | 1.01 to 1.52 |

***p<0.05, **p<0.01, ***p<0.001, N = sample size, OR = Odds Ratio, CI = Confidence Intervals**

# **Table S2. Regression analyses of baseline variables associated with depression (N = 11,515)**

|  |  | **Attrition** |  |
| --- | --- | --- | --- |
|  |  | **β** | **95% CI** |
| Mental health | Anxiety | 0.48*** | 0.47 to 0.50 |
|  | PTSD | 0.18*** | 0.16 to 0.19 |
| Frequency of alcohol use | Never drank | 0.00 |  |
|  | Less than 4x a week | 0.01 | -0.05 to 0.07 |
|  | 4+ times a week | 0.04 | -0.05 to 0.13 |
| At-risk alcohol use | Non-drinkers/low risk | 0.00 |  |
|  | At-risk drinkers | -0.01 | -0.09 to 0.06 |
| COVID-19 infection | No | 0.00 |  |
|  | Yes | -0.01 | -0.10 to 0.05 |
|  | Unsure | -0.02 | -0.06 to 0.04 |
| Bereavement | No | 0.00 |  |
|  | Yes | 0.03 | -0.01 to 0.07 |
| Work stressors | Work stressors | 0.02* | 0.00 to 0.03 |
| Discrimination | No discrimination | 0.00 |  |
|  | From patients/public | 0.11*** | 0.05 to 0.17 |
|  | From other staff | 0.14*** | 0.08 to 0.20 |
| Gender | Male | 0.00 |  |
|  | Female | -0.15*** | -0.20 to -0.10 |
| Age | Age >35 | 0.00 |  |
|  | Age 36-45 | -0.09** | -0.15 to -0.04 |
|  | Age 46-55 | -0.13*** | -0.19 to -0.07 |
|  | Age 55+ | -0.21*** | -0.28 to -0.15 |
| Marital status | Single | 0.00 |  |
|  | Living with partner | -0.17*** | -0.24 to -0.10 |
|  | Married | -0.21*** | -0.27 to -0.15 |
|  | Divorced/separated | -0.06 | -0.15 to 0.03 |
|  | Widowed | -0.13 | -0.33 to 0.08 |
| Education | A-Level or below | 0.00 |  |
|  | Undergraduate degree | -0.09** | -0.15 to -0.04 |
|  | Postgraduate degree | -0.10** | -0.16 to -0.04 |
| Ethnicity | White British | 0.00 |  |
|  | Any other White background | 0.00 | -0.08 to 0.08 |
|  | Indian | -0.11** | -0.19 to -0.03 |
|  | Pakistani & Bangladeshi | -0.04 | -0.16 to 0.08 |
|  | Any other Asian background | -0.12* | -0.22 to -0.02 |
|  | Black | -0.24*** | -0.35 to -0.13 |
|  | Mixed | -0.04 | -0.13 to 0.06 |
|  | Any other ethnic group | -0.05 | -0.20 to 0.10 |
| Country of birth | UK born | 0.00 |  |
|  | Born elsewhere | 0.05 | -0.01 to 0.11 |
| Job role | Medical role | 0.00 |  |
|  | Nursing | 0.22*** | 0.15 to 0.28 |
|  | Allied health professional | 0.12*** | 0.07 to 0.17 |
|  | Dental | 0.17*** | 0.08 to 0.26 |
|  | Administrative | 0.23*** | 0.14 to 0.33 |

***p<0.05, **p<0.01, ***p<0.001, N = sample size, β = Beta Coefficient, CI = Confidence Intervals**

# **Table S3. Regression analyses of baseline variables associated with anxiety (N = 11,515)**

|  | . | **Attrition** |  |
| --- | --- | --- | --- |
|  |  | **β** | **95% CI** |
| Mental health | Anxiety | 0.59*** | 0.57 to 0.60 |
|  | PTSD | 0.23*** | 0.21 to 0.24 |
| Frequency of alcohol use | Never drank | 0.00 |  |
|  | Less than 4x a week | 0.03 | -0.03 to 0.11 |
|  | 4+ times a week | 0.11* | 0.00 to 0.88 |
| At-risk alcohol use | Non-drinkers/low risk | 0.00 |  |
|  | At-risk drinkers | 0.03 | -0.05 to 0.11 |
| COVID-19 infection | No | 0.00 |  |
|  | Yes | 0.02 | -0.04 to 0.07 |
|  | Unsure | 0.10* | 0.01 to 0.18 |
| Bereavement | No | 0.00 |  |
|  | Yes | 0.00 | -0.04 to 0.05 |
| Work stressors | Work stressors | 0.04*** | 0.03 to 0.06 |
| Discrimination | No discrimination | 0.00 |  |
|  | From patients/public | 0.05 | -0.01 to 0.12 |
|  | From other staff | 0.08* | 0.02 to 0.15 |
| Gender | Male | 0.00 |  |
|  | Female | 0.29*** | 0.23 to 0.34 |
| Age | >35 | 0.00 |  |
|  | 36-45 | -0.09** | -0.16 to -0.03 |
|  | 46-55 | -0.18*** | -0.24 to -0.11 |
|  | 55+ | -0.27*** | -0.34 to -0.20 |
| Marital status | Single | 0.00 |  |
|  | Living with partner | 0.07 | -0.00 to 0.14 |
|  | Married | 0.05 | -0.01 to 0.11 |
|  | Divorced/separated | -0.07 | -0.17 to 0.02 |
|  | Widowed | -0.13 | -0.31 to 0.14 |
| Education | A-Level or below | 0.00 |  |
|  | Undergraduate degree | 0.07* | 0.00 to 0.13 |
|  | Postgraduate degree | 0.08* | 0.00 to 0.14 |
| Ethnicity | White British | 0.00 |  |
|  | Any other White background | 0.02 | -0.07 to 0.11 |
|  | Indian | -0.04 | -0.13 to 0.05 |
|  | Pakistani & Bangladeshi | -0.09 | -0.22 to 0.05 |
|  | Any other Asian background | -0.02 | -0.13 to 0.09 |
|  | Black | -0.13* | -0.25 to -0.02 |
|  | Mixed | -0.07 | -0.18 to 0.04 |
|  | Any other ethnic group | 0.01 | -0.15 to 0.18 |
| Country of birth | UK born | 0.00 |  |
|  | Born elsewhere | -0.07* | -0.14 to -0.00 |
| Job role | Medical role | 0.00 |  |
|  | Nursing | -0.09** | -0.17 to -0.02 |
|  | Allied health professional | -0.08* | -0.13 to -0.02 |
|  | Dental | -0.02 | -0.12 to 0.08 |
|  | Administrative | -0.14* | -0.24 to -0.03 |

***p<0.05, **p<0.01, ***p<0.001, N = sample size, β = Beta Coefficient, CI = Confidence Intervals**

# **Table S4. Regression analyses of baseline variables associated with PTSD (N = 11,515)**

|  |  | **Attrition** |  |
| --- | --- | --- | --- |
|  |  | **β** | **95% CI** |
| Mental health | Depression | 0.37*** | 0.35 to 0.40 |
|  | Anxiety | 0.35*** | 0.33 to 0.38 |
| Frequency of alcohol use | Never drank | 0.00 |  |
|  | Less than 4x a week | -0.08 | -0.17 to 0.00 |
|  | 4+ times a week | -0.05 | -0.18 to 0.07 |
| At-risk alcohol use | Non-drinkers/low risk | 0.00 |  |
|  | At-risk drinkers | 0.03 | -0.07 to 0.14 |
| COVID-19 infection | No | 0.00 |  |
|  | Yes | 0.05 | -0.02 to 0.12 |
|  | Unsure | 0.06 | -0.05 to 0.17 |
| Bereavement | No | 0.00 |  |
|  | Yes | 0.14*** | 0.09 to 0.20 |
| Work stressors | Work stressors | 0.00 | -0.02 to 0.02 |
| Discrimination | No discrimination | 0.00 |  |
|  | From patients/public | 0.24*** | 0.16 to 0.32 |
|  | From other staff | 0.57*** | 0.49 to 0.65 |
| Gender | Male | 0.00 |  |
|  | Female | 0.10** | 0.03 to 0.17 |
| Age | >35 | 0.00 |  |
|  | 36-45 | 0.08* | 0.00 to 0.16 |
|  | 46-55 | 0.05 | -0.03 to 0.14 |
|  | 55+ | 0.12* | 0.03 to 0.21 |
| Marital status | Single | 0.00 |  |
|  | Living with partner | 0.01 | -0.08 to 0.11 |
|  | Married | -0.04 | -0.12 to 0.04 |
|  | Divorced/separated | 0.25*** | 0.13 to 0.38 |
|  | Widowed | 0.48** | 0.19 to 0.77 |
| Education | A-Level or below | 0.00 |  |
|  | Undergraduate degree | -0.15*** | -0.24 to -0.07 |
|  | Postgraduate degree | -0.22*** | -0.31 to -0.13 |
| Ethnicity | White British | 0.00 |  |
|  | Any other White background | 0.06 | -0.05 to 0.17 |
|  | Indian | 0.25*** | 0.13 to 0.26 |
|  | Pakistani & Bangladeshi | 0.25** | 0.08 to 0.42 |
|  | Any other Asian background | 0.13 | -0.01 to 0.27 |
|  | Black | 0.15 | -0.00 to 0.30 |
|  | Mixed | 0.14* | 0.00 to 0.28 |
|  | Any other ethnic group | 0.23* | 0.02 to 0.44 |
| Country of birth | UK born | 0.00 |  |
|  | Born elsewhere | 0.00 | -0.09 to 0.09 |
| Job role | Medical role | 0.00 |  |
|  | Nursing | 0.22*** | 0.15 to 0.28 |
|  | Allied health professional | 0.12*** | 0.07 to 0.17 |
|  | Dental | 0.17*** | 0.08 to 0.26 |
|  | Administrative | 0.23*** | 0.14 to 0.33 |

***p<0.05, **p<0.01, ***p<0.001, N = sample size, β = Beta Coefficient, CI = Confidence Intervals**

# **Table S5. Final regression model of baseline variables associated with both attrition and mental health (N = 11,695)**

|  |  | **Attrition** |  |
| --- | --- | --- | --- |
|  |  | **OR** | **95% CI** |
| Mental health | Depression | 1.07** | 1.03 to 1.11 |
|  | Anxiety | 0.96* | 0.93 to 0.99 |
|  | PTSD | 1.05** | 1.02 to 1.08 |
| Bereavement | No bereavement | 1.00 |  |
|  | Bereavement | 1.12** | 1.03 to 1.22 |
| Gender | Male | 1.00 |  |
|  | Female | 1.13* | 1.03 to 1.25 |
| Age | >35 | 1.00 |  |
|  | 36-45 | 0.86* | 0.76 to 0.98 |
|  | 46-55 | 0.80** | 0.70 to 0.91 |
|  | 55+ | 0.70*** | 0.61 to 0.80 |
| Marital status | Single | 1.00 |  |
|  | Living with partner | 1.19* | 1.03 to 1.38 |
|  | Married | 1.08 | 0.96 to 1.23 |
|  | Divorced/separated | 1.25* | 1.03 to 1.51 |
|  | Widowed | 1.31 | 0.85 to 2.02 |
| Education | A-Level or below | 1.00 |  |
|  | Undergraduate degree | 0.92 | 0.81 to 1.04 |
|  | Postgraduate degree | 0.82** | 0.72 to 0.93 |
| Ethnicity | White British | 1.00 |  |
|  | Any other White background | 0.87 | 0.73 to 1.03 |
|  | Indian | 1.40*** | 1.18 to 1.66 |
|  | Pakistani & Bangladeshi | 1.34* | 1.03 to 1.74 |
|  | Any other Asian background | 0.92 | 0.75 to 1.13 |
|  | Black | 1.37* | 1.07 to 1.74 |
|  | Mixed | 1.08 | 0.88 to 1.33 |
|  | Any other ethnic group | 1.69** | 1.19 to 2.39 |
| Country of birth | UK born | 1.00 |  |
|  | Born elsewhere | 1.44*** | 1.26 to 1.64 |
| Job role | Medical role | 1.00 |  |
|  | Nursing | 1.19* | 1.04 to 1.36 |
|  | Allied health professional | 1.32*** | 1.18 to 1.48 |
|  | Dental | 1.36** | 1.12 to 1.65 |
|  | Administrative | 1.24* | 1.01 to 1.51 |

***p<0.05, **p<0.01, ***p<0.001, N = sample size, OR = odds ratio, CI = Confidence Intervals**

# **Table S6. Descriptive statistics and missing data for level one predictor variables (vary by time point) at each time point, for participants who completed at least two surveys (N = 6,973). Percentages are weighted.**

|  |  | **Baseline** | **6-month follow up** | **10-month follow up** |
| --- | --- | --- | --- | --- |
|  |  | **N (%)** | **N (%)** | **N (%)** |
| Access to PPE ^a^ | |  |  |  |
|  | All/Most of the time | 6044 (91.04) | 4366 (88.94) | 4453 (87.68) |
|  | Some of the time | 79 (1.20) | 58 (1.18) | 71 (1.42) |
|  | Rarely/not at all | 58 (0.86) | 37 (0.74) | 49 (0.93) |
|  | Not applicable | 438 (6.64) | 416 (8.50) | 478 (9.51) |
|  | *Missing* | *17 (0.26)* | *33 (0.64)* | *23 (0.46)* |
| Previous infection | |  |  |  |
|  | No | 5200 (74.74) | 3879 (58.88) | 3643 (54.75) |
|  | Yes | 1339 (19.02) | 1132 (17.03) | 1642 (24.33) |
|  | Unsure | 422 (6.09) | 215 (3.24) | 235 (3.53) |
|  | Prefer not to say | 2 (0.02) | 0 (0.00) | 1 (0.01) |
|  | *Missing* | *10 (0.12)* | *1747 (20.85)* | *1452 (17.38)* |
| C19 bereavement | |  |  |  |
|  | No | 3913 (55.94) | 2982 (45.02) | 3071 (45.76) |
|  | Yes | 3056 (43.00) | 2260 (34.31) | 2457 (36.92) |
|  | Prefer not to say | 4 (0.06) | 4 (0.06) | 4 (0.06) |
|  | *Missing* | *0 (0.00)* | *1727 (20.61)* | *1441 (17.25)* |
| Feel secure raising concerns ^a^ | |  |  |  |
|  | Agree | 5495 (82.76) | 4078 (83.18) | 4149 (81.81) |
|  | Neither agree nor disagree | 454 (6.81) | 334 (6.79) | 344 (6.82) |
|  | Disagree | 473 (7.20) | 358 (7.22) | 438 (8.54) |
|  | Not applicable | 190 (2.89) | 94 (1.94) | 98 (1.95) |
|  | *Missing* | *24 (0.35)* | *46 (0.87)* | *45 (0.88)* |
| Organisation would address concerns ^a^ | |  |  |  |
|  | Agree | 4625 (69.71) | 3470 (70.67) | 3335 (65.66) |
|  | Neither agree nor disagree | 1097 (16.57) | 813 (16.56) | 860 (17.10) |
|  | Disagree | 750 (11.24) | 498 (10.20) | 755 (14.82) |
|  | Not applicable | 144 (2.19) | 81 (1.65) | 84 (1.64) |
|  | *Missing* | *20 (0.29)* | *48 (0.92)* | *40 (0.78)* |
| Discrimination at work ^a^ | |  |  |  |
|  | No discrimination | 4707 (71.23) | 4059 (83.31) | 3962 (78.75) |
|  | From patients/public | 903 (13.80) | 288 (5.93) | 398 (7.84) |
|  | From other staff | 864 (13.09) | 470 (9.60) | 617 (12.18) |
|  | *Missing* | *124 (1.87)* | *57 (1.16)* | *62 (1.22)* |

^a^ Analyses restricted to those working at the time

Baseline working N = 6,636; 6-month follow-up working N = 4,910; 10-month follow-up working N = 5,07

# **Table S7. Characteristics of alcohol use and mental health at each time point. Percentages are weighted to account for attrition at follow up.**

|  |  | **Baseline** | **6-month follow up** | **10-month follow up** |
| --- | --- | --- | --- | --- |
|  |  | **N (%)**  **N = 6,973** | **N (%)**  **N = 5,164** | **N (%)**  **N = 5,454** |
| Frequency of alcohol use | |  |  |  |
|  | Never | 1,035 (15.03) | - | - |
|  | < 4 times a week | 5,101 (73.08) | - | - |
|  | 4+ times a week | 808 (11.47) | - | - |
|  | Prefer not to say | 14 (0.21) | - | - |
|  | *Missing* | *15 (0.21)* | - | - |
| Change in alcohol use | |  |  |  |
|  | Never drank | 1,035 (15.03) | 811 (15.67) | 742 (13.71) |
|  | Has not changed | 3,496 (50.23) | 2,455 (47.78) | 3,072 (56.56) |
|  | Drink less often | 920 (13.07) | 1,186 (22.86) | 1,061 (19.28) |
|  | Drink more often | 1,494 (21.25) | 697 (13.42) | 564 (10.19) |
|  | Prefer not to say | 0 (0.00) | 12 (0.22) | 10 (0.17) |
|  | *Missing* | *28 (0.42)* | *3 (0.05)* | *5 (0.09)* |
| At-risk alcohol consumption | |  |  |  |
|  | Non-drinkers/low risk | 6,119 (87.99) | - | - |
|  | At-risk drinkers | 821 (11.53) | - | - |
|  | Prefer not to say | 29 (0.42) | - | - |
|  | *Missing* | *4 (0.05)* | *-* | *-* |
| Mental health (Mean ±SD) | |  |  |  |
|  | Depression (PHQ-2) | 1.07 (1.49) | 0.95 (1.43) | 0.94 (1.43) |
|  | *Missing* | *86 (1.15)* | *43 (0.82)* | *31 (0.54)* |
|  | Anxiety (GAD-2) | 1.45 (1.67) | 1.35 (1.59) | 1.37 (1.62) |
|  | *Missing* | *45 (0.60)* | *25 (0.50)* | *26 (0.44)* |
|  | PTSD (PCL-C) | 3.35 (1.84) | 3.30 (1.83) | 3.28 (1.79) |
|  | *Missing* | *18 (0.24)* | *28 (0.60)* | *22 (0.53)* |

# **Table S8. Prevalence of alcohol use and mental health at each time point for those with completed all three surveys (N = 3,645). Percentages are weighted to account for attrition at follow up.**

|  |  | **Baseline** | **6-month follow up** | **10-month follow up** |
| --- | --- | --- | --- | --- |
|  |  | **N (%)** | **N (%)** | **N (%)** |
| Frequency of alcohol use | |  |  |  |
|  | Never | 525 (14.38) | - | - |
|  | < 4 times a week | 2,666 (73.18) | - | - |
|  | 4+ times a week | 441 (12.08) | - | - |
|  | Prefer not to say | 6 (0.17) | - | - |
|  | *Missing* | *7 (0.19)* | - | - |
| Change in alcohol use | |  |  |  |
|  | Never drank | 525 (14.38) | 541 (14.80) | 493 (13.46) |
|  | Has not changed | 1,895 (52.00) | 1,797 (49.33) | 2,122 (58.27) |
|  | Drink less often | 460 (12.61) | 824 (22.61) | 675 (18.54) |
|  | Drink more often | 750 (20.59) | 475 (13.04) | 348 (9.54) |
|  | Prefer not to say | 0 (0.00) | 6 (0.17) | 3 (0.08) |
|  | *Missing* | *15 (0.41)* | *2 (0.05)* | *4 (0.11)* |
| At-risk alcohol consumption | |  |  |  |
|  | Non-drinkers/low risk | 3,208 (88.01) | - | - |
|  | At-risk drinkers | 421 (11.54) | - | - |
|  | Prefer not to say | 14 (0.39) | - | - |
|  | *Missing* | *2 (0.05)* | *-* | *-* |
| Mental health (Mean ±SD) | |  |  |  |
|  | Depression (PHQ-2) | 0.98 (1.43) | 0.90 (1.38) | 0.92 (1.41) |
|  | *Missing* | *33 (0.73)* | *26 (0.72)* | *21 (0.58)* |
|  | Anxiety (GAD-2) | 1.40 (1.65) | 1.31 (1.58) | 1.35 (1.61) |
|  | *Missing* | *20 (0.44)* | *16 (0.43)* | *16 (0.44)* |
|  | PTSD (PCL-C) | 3.25 (1.74) | 3.24 (1.77) | 3.23 (1.75) |
|  | *Missing* | *7 (0.16)* | *20 (0.54)* | *16 (0.44)* |
