# Supplementary figures and images for: Longitudinal associations between alcohol use, occupational stressors, and mental health among healthcare and ancillary workers in the United Kingdom during the COVID-19 pandemic (UK-REACH)
Source: BMC Med. 2025 Nov 28;23:665. doi: 10.1186/s12916-025-04474-4 (PMC12664230; doi:10.1186/s12916-025-04474-4)

Supplementary Materials

[**Figure S1. Participant flow chart.** 2](#_Toc211862914)

**
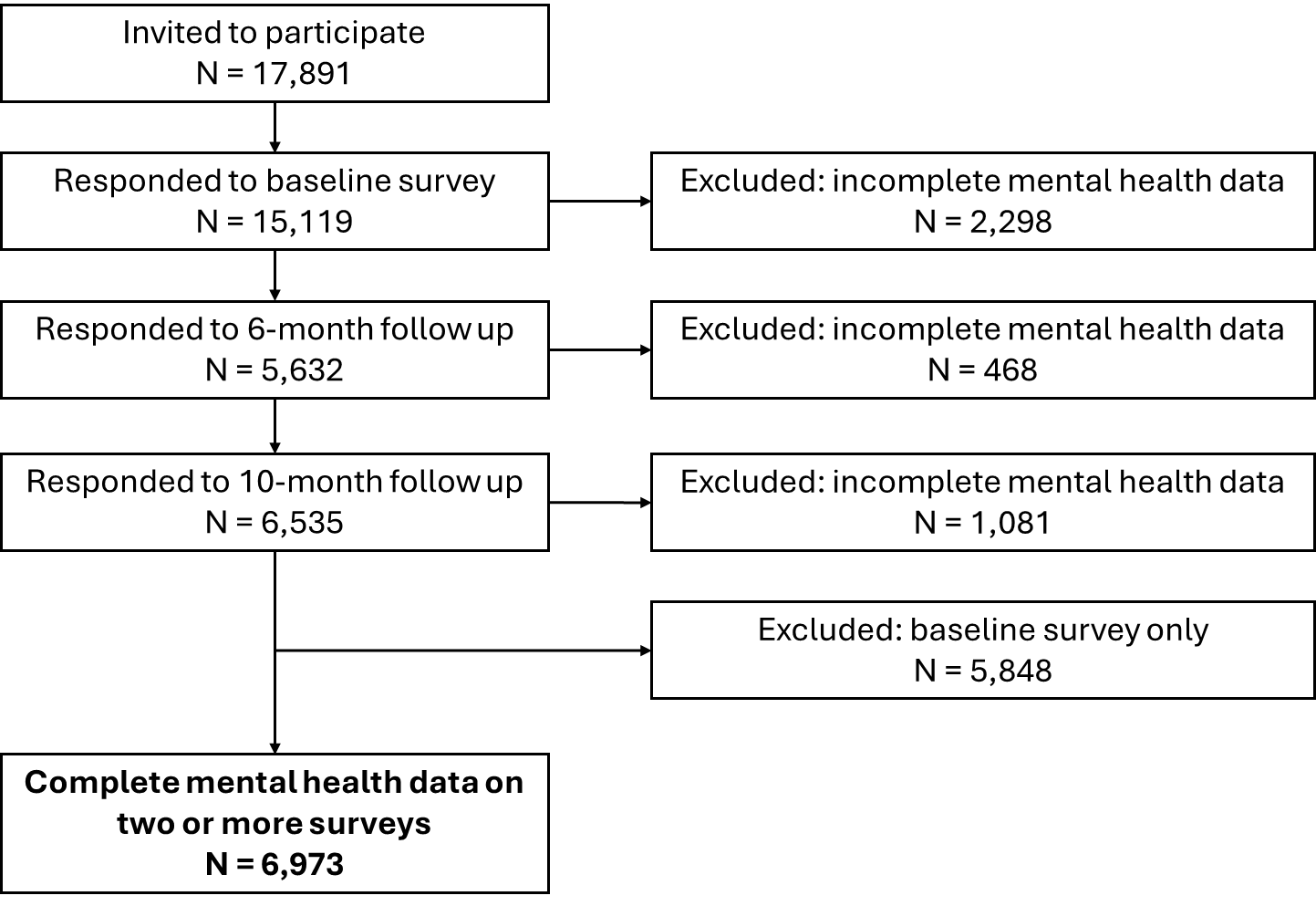
**

# **Figure S1. Participant flow chart.**

Supplement: Supplementary file 2 — Additional file 2. Figure S1. Figure S1. Participant flow diagram [file 12916_2025_4474_MOESM2_ESM.docx]
