# Supplementary material for: Longitudinal associations between alcohol use, occupational stressors, and mental health among healthcare and ancillary workers in the United Kingdom during the COVID-19 pandemic (UK-REACH)
Source: BMC Med. 2025 Nov 28;23:665. doi: 10.1186/s12916-025-04474-4 (PMC12664230; doi:10.1186/s12916-025-04474-4)
Supplement: Supplementary file 3 — Additional file 3. [file 12916_2025_4474_MOESM3_ESM.docx]

STROBE Statement—Checklist of items that should be included in reports of ***cohort studies***

|  | Item No | Recommendation | Page No |
| --- | --- | --- | --- |
| **Title and abstract** | 1 | (*a*) Indicate the study’s design with a commonly used term in the title or the abstract | (a) 1, lines 3-5 |
|  |  | (*b*) Provide in the abstract an informative and balanced summary of what was done and what was found | (b) 3, lines 45-67 |
| Introduction | | | |
| Background/rationale | 2 | Explain the scientific background and rationale for the investigation being reported | 4-5, lines 70-92 |
| Objectives | 3 | State specific objectives, including any prespecified hypotheses | 5, lines 93-116 |
| Methods | | | |
| Study design | 4 | Present key elements of study design early in the paper | 6, lines 119-121 |
| Setting | 5 | Describe the setting, locations, and relevant dates, including periods of recruitment, exposure, follow-up, and data collection | 6, lines 121-146 |
| Participants | 6 | (*a*) Give the eligibility criteria, and the sources and methods of selection of participants. Describe methods of follow-up | (a) 6-7, lines 119-146 |
|  |  | (*b*) For matched studies, give matching criteria and number of exposed and unexposed | (b) N/A |
| Variables | 7 | Clearly define all outcomes, exposures, predictors, potential confounders, and effect modifiers. Give diagnostic criteria, if applicable | 7-9, lines 148-190 |
| Data sources/ measurement | 8* | For each variable of interest, give sources of data and details of methods of assessment (measurement). Describe comparability of assessment methods if there is more than one group | 7-9, lines 148-190 |
| Bias | 9 | Describe any efforts to address potential sources of bias | 10, lines 217-224 |
| Study size | 10 | Explain how the study size was arrived at | 6, lines 135-142 |
| Quantitative variables | 11 | Explain how quantitative variables were handled in the analyses. If applicable, describe which groupings were chosen and why | 7-9, lines 148-190 |
| Statistical methods | 12 | (*a*) Describe all statistical methods, including those used to control for confounding | (a) 9, lines 190-215 |
|  |  | (*b*) Describe any methods used to examine subgroups and interactions | (b) 9, lines 207-208 |
|  |  | (*c*) Explain how missing data were addressed | (c) 10, lines 217-224 |
|  |  | (*d*) If applicable, explain how loss to follow-up was addressed | (d) 10, lines 217-224 |
|  |  | (*e*) Describe any sensitivity analyses | (e) N/A |
| Results | | |  |
| Participants | 13* | (a) Report numbers of individuals at each stage of study—eg numbers potentially eligible, examined for eligibility, confirmed eligible, included in the study, completing follow-up, and analysed | (a) 11, lines 236-242 (Figure S1) |
|  |  | (b) Give reasons for non-participation at each stage | (b) N/A |
|  |  | (c) Consider use of a flow diagram | (c) Figure S1 |
| Descriptive data | 14* | (a) Give characteristics of study participants (eg demographic, clinical, social) and information on exposures and potential confounders | (a) Table 1  (b) Table S6 & S7)  (c) N/A |
|  |  | (b) Indicate number of participants with missing data for each variable of interest |  |
|  |  | (c) Summarise follow-up time (eg, average and total amount) |  |
| Outcome data | 15* | Report numbers of outcome events or summary measures over time | Table S7&8 |

| Main results | 16 | (*a*) Give unadjusted estimates and, if applicable, confounder-adjusted estimates and their precision (eg, 95% confidence interval). Make clear which confounders were adjusted for and why they were included | (a) 11-13, lines 245-287, Tables 3-5.  (b) N/A |
| --- | --- | --- | --- |
|  |  | (*b*) Report category boundaries when continuous variables were categorized | (c) N/A |
|  |  | (*c*) If relevant, consider translating estimates of relative risk into absolute risk for a meaningful time period |  |
| Other analyses | 17 | Report other analyses done—eg analyses of subgroups and interactions, and sensitivity analyses | 13, lines 290-295 |
| Discussion | | | |
| Key results | 18 | Summarise key results with reference to study objectives | 13, lines 297-310 |
| Limitations | 19 | Discuss limitations of the study, taking into account sources of potential bias or imprecision. Discuss both direction and magnitude of any potential bias | 15, lines 340-352 |
| Interpretation | 20 | Give a cautious overall interpretation of results considering objectives, limitations, multiplicity of analyses, results from similar studies, and other relevant evidence | 13-16, lines 297-369 |
| Generalisability | 21 | Discuss the generalisability (external validity) of the study results | 15, lines 348-352 |
| Other information | | | |
| Funding | 22 | Give the source of funding and the role of the funders for the present study and, if applicable, for the original study on which the present article is based | 17, lines 393-407 |

*Give information separately for exposed and unexposed groups.

**Note:** An Explanation and Elaboration article discusses each checklist item and gives methodological background and published examples of transparent reporting. The STROBE checklist is best used in conjunction with this article (freely available on the Web sites of PLoS Medicine at http://www.plosmedicine.org/, Annals of Internal Medicine at http://www.annals.org/, and Epidemiology at http://www.epidem.com/). Information on the STROBE Initiative is available at http://www.strobe-statement.org.
